# Evaluation of retinectomy in the treatment of severe proliferative vitreoretinopathy

**DOI:** 10.1186/s40942-015-0018-3

**Published:** 2015-10-08

**Authors:** Thaís Sousa Mendes, André Marcelo Vieira Gomes, Bruno Saraiva Rocha, Hélcio Valério Passos Junior, Suel Abujamra

**Affiliations:** 1 Department of Ophthalmology, Suel Abujamra Institute, 693 Tamandare Street, 9th floor, Sao Paulo, SP 01531-000 Brazil; 2 Department of Ophthalmology, University of Sao Paulo, Sao Paulo, SP 05403-900 Brazil

**Keywords:** Retinal detachment, Retinectomy, Severe proliferative vitreoretinopathy

## Abstract

**Background:**

To evaluate the postoperative results and efficacy of retinectomy in the treatment of severe proliferative vitreoretinopathy (PVR).

**Methods:**

The study involved 38 individuals with rhegmatogenous retinal detachment initially diagnosed with PVR grade C3 who had undergone *pars plana* vitrectomy combined with scleral buckle and silicone oil as a tamponade. A standard three-port *pars plana* vitrectomy was performed, and the extension of performing a retinectomy was decided during the procedure. The patients were followed for a minimum of 6 months after the last surgical procedure. Reoperation, postoperative hypotony and final reattachment rates were evaluated. Statistical analysis was performed with an Exact Fisher’s test.

**Results:**

The mean preoperative visual acuity was >1.3 logMAR. The postoperative visual acuity improved and ranged from 1.3 to 0.7 logMAR (*p* < 0.63). The preoperative intraocular pressure was 10.2 mmHg and postoperatively was 11.6 mmHg. Postoperative hypotony was observed in 15.8 % of the cases. The reoperation rate after the first procedure was 63.2 %. A total of 44.7 % of the patients needed a retinectomy greater than 270° for a final anatomical success. The final retinal reattachment rate was 94.7 %.

**Conclusions:**

Retinectomy can be an effective surgical procedure in the treatment of severe PVR, and it may increase the final reattachment rate.

## Background

Proliferative vitreoretinopathy (PVR) is the most common cause of failure in the management of rhegmatogenous retinal detachment (RRD). PVR occurs in 5–10 % of retinal detachment (RD) surgeries, and it comprises glial and retinal pigment epithelium cells that migrate and generate membranes usually on the lower retina as a result of gravity. Contraction of these membranes can cause new retinal tears, macular pucker, recurrent RD and hypotony. PVR categories are grades A, B, and C1–C5 [1–3].

The rate of anatomic success in RRD surgery can reach 80–90 % due to current advanced surgical techniques, especially in cases of RD involving mild PVR (grades A–C1). However, in cases of severe PVR (grades C2–C5), the prognosis is significantly poorer, considering anatomical and visual outcomes [1–4].

In the past, there has been controversy regarding the use of silicone oil versus gas as a tamponade in cases of RD associated with severe PVR. The Silicone Study (1992) concluded that perfluoropropane (C3F8) or silicone oil is more effective than SF6 in the treatment of PVR cases [5]. The use of pharmacological agents as adjuvant therapy in RD surgery has been described. Studies have described the use of daunomycin, 5-fluorouacil and heparin as adjuvant therapy. However, these studies reported that these drugs failed to improve final anatomic results [2, 6, 7].

In 1979, Machemer first described retinectomy as a treatment for complex RRD. Since that study, many authors have reported relaxing retinectomy as a valuable technique in the surgical management of severe PVR to achieve a high rate of anatomical success. Additionally, recent studies have suggested silicone oil as a superior tamponade compared to gas in eyes with PVR [4, 8].

Our study aimed to evaluate the efficacy of retinectomy in the surgical treatment of RRD associated with severe PVR and its effect on the reoperation rate and final retinal reattachment.

## Methods

A retrospective study was conducted in 38 eyes of 38 patients with RRD initially diagnosed with PVR grade C3 who had undergone *pars plana* vitrectomy (PPV) combined with scleral buckle and silicone oil as a tamponade from June 2006 to June 2010. The study excluded eyes with previous vitrectomy for RRD, non-RRD and trauma.

The surgical technique included 360° peritomy, scleral buckle, and three-port incision (scleretomies) combined with a 23-gauge vitrectomy, which was performed by one experienced retinal surgeon (AG). Retinectomy extension was performed according to the surgeon’s decision at the time of surgery based on retinal shortening (180°, 270° or 360°). For all cases, 5000 centistokes silicone oil was used as a tamponade and a scleral buckle was not placed if a 360° shortening was made necessary during vitrectomy.

The postoperative follow-up was performed at 1 day, 1 week, 15, 30, 60, 90 and 6 months after the last surgical procedure was performed. Postoperative hypotony was defined as intraocular pressure ranging from 0 to 5 mmHg. Patients who did not complete 6 months of follow-up were excluded. Silicone oil was removed after a minimum of 3 months in patients with no signs of recurrent PVR or persistent RD.

A *Fisher’s* test was used to verify the association between the rate of reoperation and retinectomy performed. The postoperative average best-corrected visual acuity (BCVA) was analyzed with a *Mann*–*Whitney* test because of non-measurable values in LogMAR. A *p* value <0.05 was considered statistically significant.

## Results

Patients were 62.9 ± 15.3 years of age and 52.5 % were male. Regarding lens status, 78.9 % of patients were phakic, 15.8 % were pseudophakic and 5.3 % were aphakic. The average preoperative IOP was 10.2 ± 4.1 mmHg and postoperative IOP was 11.6 ± 3.8 mm Hg.

The average preoperative BCVA was greater than 1.3 LogMAR (<20/400 Snellen Equivalent). All eyes achieved improvement to a final BCVA ranging from 1.3 to 0.7 LogMAR. Postoperative hypotony was determined in 15.8 % of the patients. Only one eye developed neovascular glaucoma (Table 1).

**Table 1:** Patient demographics, retinectomy extension and postoperative complications

	N	Percentage (%)
Age	62.9 (±15.3)	
Gender		
Female	20	52.5
Male	18	47.5
Lens status		
Phakic	30	78.9
Pseudophakic	6	15.8
Aphakic	2	5.3
IOP (mmHg)		
Preoperative	10.2 (±4.1)	
Postoperative	11.6 (±3.8)	
Retinectomy extension		
180°	21	55.3
270°	7	18.4
360°	10	26.3
Neovascular glaucoma	1	2.6
Postoperative hypotony	6	15.8

After the first procedure, 63.2 % of eyes needed a second surgery, including enlargement of the retinectomy from 180° to 270° (n = 11) or from 270° to 360° (n = 1), silicone oil removal (n = 10) and standard intraocular lens fixation (n = 2). A total of four eyes needed a third surgical procedure, including enlargement of the retinectomy from 270° to 360°. A final reattachment was observed in 94.7 % of the patients. Regarding extension of the retinectomy, 44.7 % eyes underwent retinectomy greater than 270° after the first surgery (Table 1).

There was an association between retinectomy extension and postoperative visual acuity. A total of 87 % (n = 33) of the eyes had better or stabilized postoperative VA compared to preoperative VA. Regarding hypotony, there was no association between the hypotony rate and retinectomy extension (Tables 2, 3, 4). No patients needed a scleral buckle removal.

**Table 2:** Preoperative and postoperative best-corrected visual acuity

BCVA					
LogMAR	Snellen	Preoperative		Postoperative	
		N	Percentage (%)	N	Percentage (%)
0.7	20/100	0	0	10	26.3
1	20/200	3	7.9	11	28.9
1.3	20/400	2	5.3	0	0
>1.3–1.6	<20/400–5/400	2	5.3	6	15.8
1.6	5/400	26	68.4	3	7.9
>1.6	<5/400	5	13.2	8	21.1

**Table 3:** Final best corrected visual acuity and retinectomy extension

	Retinectomy extension			Total
	360°	270°	180°	
Change in BCVA				
Worsening	0	3	2	5
Better or no change	10	4	19	33
Total	10	7	21	38

* *p* value = 0.049

**Table 4:** Hypotony and retinectomy extension

	Hypotony		Total
	Yes	No	
Retinectomy extension			
360°	1	9	10
270°	3	4	7
180°	2	19	21
Total	6	32	38

* *p* value = 0.13

## Discussion

Proliferative vitreoretinopathy remains the primary cause of unsuccessful RRD surgery. Relaxing retinectomies are mandatory when a complete relief of retinal traction is not possible, even after membrane removal. Previous studies reported recurrent RD after 180°–360° retinectomies from 17 to 48 %. Our study found a higher rate of recurrent RD; however, most of the studies excluded eyes with PVR greater than C2 [9, 10].

Patients with severe PVR C3 who undergo retinectomy are more likely to develop postoperative PVR because of retinal pigment epithelium exposure, proliferation and migration of inflammatory cells on the retinal surface [2, 3]. In our study, all patients were classified as PVR greater than C2. This finding may explain the higher reoperation rate after the first procedure. However, the final reattachment rate of 94.7 % was greater than reports in the literature for severe PVR cases [8, 9, 11].

Postoperative hypotony is a common complication after large retinectomy and previous studies have reported a 15–40 % rate in eyes that underwent a 360° retinectomy [10–13]. Additionally, Teke et al. [9] reported 1.7 % phthisical eyes. We report a 15.8 % postoperative hypotony rate, but no patients developed *phthisis bulbi* in our study. This finding may be explained by our choice of silicone oil as a tamponade. Several authors believe that silicone oil may prevent hypotony and phthisis after vitrectomy [10–12, 14].

Our study has several limitations. This research is a retrospective study with a small number of patients. Additionally, we did not compare our patients with a control group in which retinectomy was not performed during the first surgical procedure. However, to the best of our knowledge, there are few studies in the literature evaluating the efficacy of retinectomy in eyes with PVR grade C3. Most studies have included PVR grade C1–C2 only [8, 9, 11]. A total of 44.7 % of the patients needed a retinectomy greater than 270° since the first surgery; nevertheless, most of them had an improved or stabilized final vision compared to preoperative VA. In the literature, only a small number of patients achieved ambulatory vision after a large retinectomy [10, 11].

## Conclusions

Proliferative vitreoretinopathy is still a major cause of failure of RRD surgery, and occasionally, multiple procedures are required when reattachment is not obtained [2, 3, 15]. Retinectomy may result in a higher anatomical success rate for severe PVR cases by relaxing the shortened retina and may not be related to worse results concerning final visual acuity or hypotony.
